# Natural Insect and Plant Micro-/Nanostructsured Surfaces: An Excellent Selection of Valuable Templates with Superhydrophobic and Self-Cleaning Properties

**DOI:** 10.3390/molecules190913614

**Published:** 2014-09-02

**Authors:** Song Ha Nguyen, Hayden K. Webb, Peter J. Mahon, Russell J. Crawford, Elena P. Ivanova

**Affiliations:** Faculty of Science, Engineering and Technology, Swinburne University of Technology, PO Box 218, Hawthorn, VIC 3122, Australia; E-Mails: songhathinguyen@swin.edu.au (S.H.N.); hkwebb@swin.edu.au (H.K.W.); pmahon@swin.edu.au (P.J.M.); rcrawford@swin.edu.au (R.J.C.)

**Keywords:** superhydrophobicity, insects, plants, cuticle, nanostructure

## Abstract

Insects and plants are two types of organisms that are widely separated on the evolutionary tree; for example, plants are mostly phototrophic organisms whilst insects are heterotrophic organisms. In order to cope with environmental stresses, their surfaces have developed cuticular layers that consist of highly sophisticated structures. These structures serve a number of purposes, and impart useful properties to these surfaces. These two groups of organisms are the only ones identified thus far that possess truly superhydrophobic and self-cleaning properties. These properties result from their micro- and nano-scale structures, comprised of three-dimensional wax formations. This review analyzes the surface topologies and surface chemistry of insects and plants in order to identify the features common to both organisms, with particular reference to their superhydrophobic and self-cleaning properties. This information will be valuable when determining the potential application of these surfaces in the design and manufacture of superhydrophobic and self-cleaning devices, including those that can be used in the manufacture of biomedical implants.

## 1. Introduction

The term “waterproofing” was first developed in the 1900s and usually refers to the application of a hydrophobic film via spraying to light-weight woven or knitted fabrics. The scientific and technical principles that are used to describe the wetting behaviors of water droplets that come into contact with solid surfaces have been extensively reviewed [1,2,3]. Whenever processes occur that involve a liquid moving onto a solid surface, there are three different interfacial boundaries involved; the solid-liquid, solid-air, and liquid-air interfaces. When scientific interest in wetting behavior first began to grow, there was an indistinguishable line between the actual surface and the “geometric surface” or projected surface. This led to inaccuracies in calculations designed to determine the wettability of solid substrates. In 1936, Wenzel was the first to propose a model in which the actual surface area of the surface was considered when identifying the extent of wettability [1]. In this work, various synthetic surfaces were prepared using different materials that contained different surface textures and their water contact angles were measured as a function of time.

In 1936, Fogg published his observation that very high water contact angles (WCA) would form on the upper surface of the leaves of *Triticum* (wheat) plants. This represented the first reported natural surface that possessed WCA above 150°. This property is now commonly known as “superhydrophobicity” [4]. Since then, nature has been the source of many valuable templates used in the design of synthetic hydrophobic materials. Shark skin, bird feathers, gecko feet, plant leaves and insects are some examples which have been shown to exhibit highly hydrophobic properties. A synthetic replica of shark skin, which formed a WCA of 146°, has been used as a model for the reduction of drag in fluid flow environments [5,6]. Gecko feet (WCA of 128° at the setae) are known to exhibit reversible adhesion with surfaces [7,8,9]. Duck feathers and those of other birds (WCA from 114° to 126°) possess corrugated surfaces that entrap air pockets that prevent water from touching the surface. These have been used as a model for water repellency treatments [3,10,11]. Each of these surfaces exhibited a WCA lower than 150°, and as such are not considered superhydrophobic. From the published literature, it appears that plant leaves and the surfaces of some insects are the only natural surfaces that exhibit both superhydrophobic and self-cleaning properties.

Even though natural non-wetting surfaces have been observed since 1936, it was only in 1997 that Barthlott introduced the archetype “lotus effect”, where water droplets contacting the top surface of lotus leaves was described in detail. Since then, naturally occurring surfaces that exhibit superhydrophobic behavior have received greater attention from both scientists and industry [12]. Taro leaves, Indian canna leaves and rice leaves are just some examples of other plants that have been identified to possess similar surface properties to that of the lotus leaves [13,14]. Insects, particularly dragonflies, damselflies and cicadas exist in the same or similar environmental conditions to these plants. This often necessitates the need for these insects to develop strategies for coping with environmental stresses such as rain drops and dirt [15,16].

### The Concept of Wettability

When a surface exhibits a high water contact angle (WCA > 150°) and low contact angle hysteresis (CAH < 10°), the surfaces are regarded as being not only superhydrophobic, but also self-cleaning [6,16,17]. CAH is the difference between the advancing and receding contact angles [18,19]. Self-cleaning occurs when the water droplets form an almost spherical shape on the surface and therefore will readily move when subjected to low tilting angles. Any dirt or contaminants on the surface are then collected by the droplets and are removed as the droplet rolls off the surface. This characteristic is usually associated with low-drag [6] and low adhesion properties [20]. Depending on the structure and chemical composition of the surface, antibiofouling [21], anisotropic wetting [14], anti-reflection [22], and anti-icing [20] properties may also be present.

Whilst Wenzel’s theory explains that surface hydrophobicity is a function of surface roughness as well as chemical composition [1], it is the theory developed by Cassie and Baxter [15] that is most often applicable for superhydrophobic surfaces. The Cassie-Baxter model also considers the topography and chemistry of a surface in determining the hydrophobicity [15]. According to this model, surface hydrophobicity is a function of surface chemical heterogeneity according to the following equation:

cos *θ* = *f*_1_ cos *θ*_1_ + *f*_2_ cos *θ*_2_ (1)

where *θ* is the observed contact angle of the heterogeneous surface, *f*_1_ and *f*_2_ are the area fractions of surface components 1 and 2, with θ_1_ and *θ*_2_ being their respective contact angles. In the case of superhydrophobic surfaces, one of the components is typically air, which exhibits a water contact angle of 180°. This allows the simplification of Equation (1) to:

cos *θ* = *f*_1_(cos *θ*_1_ + 1) − 1 (2)

Superhydrophobicity arises from the combination of hierarchical surface structures that enable the entrapment of air on low surface energy materials. The sliding angle, another parameter that is important in determining hydrophobicity, is defined as the critical angle at which the water droplets start to slide along a tilted surface [15,23,24]. A superhydrophobic surface would effectively form a composite interface (CI) with air residing between the asperities on the surface. The CI can be destabilized and irreversibly transformed into a homogeneous interface, for example, by the application of sonication [25]. There are three factors which can destroy a CI; (i) a capillary wave formed at the liquid-air interface; (ii) nanodroplet accumulation in the valleys on the surface and (iii) hydrophilic surface regions arising from the chemical heterogeneity of the surface. Hierarchical roughness or “Cassie-Baxter structure” needs to exist in order for the CI to be stabilized [25,26].

**Figure 1:**
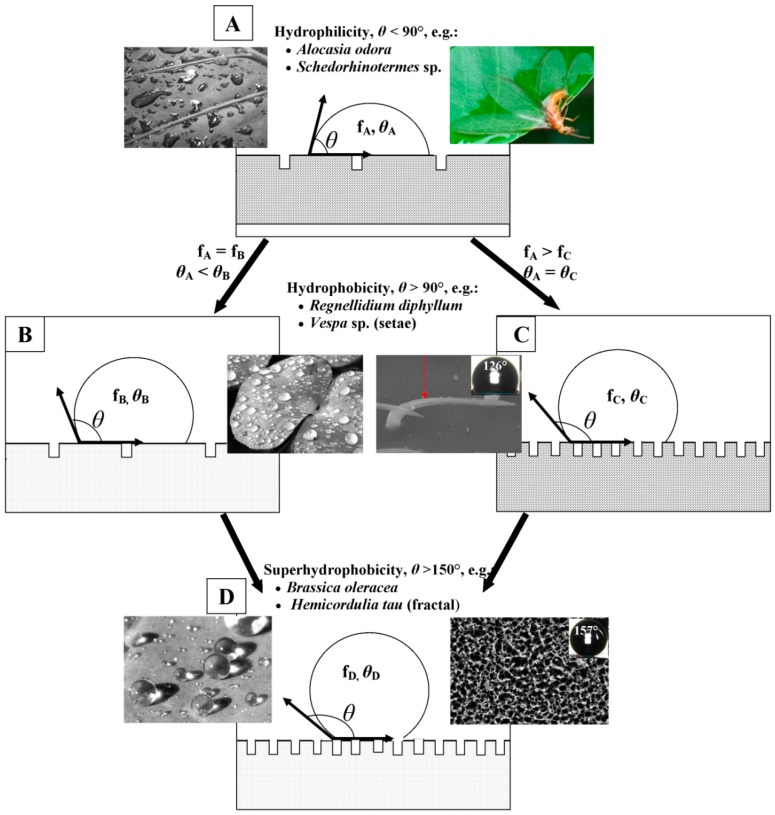
The role of topography and surface chemistry in determining (**A**) hydrophilicity, (**B**,**C**) hydrophobicity, and (**D**) superhydrophobicity of a surface, according to the Cassie-Baxter model. The surface presented in A exhibits a relatively low water contact angle (WCA), as it is composed of a material that is hydrophilic, and does not possess a surface structure that can trap significant quantities of air. Changing the chemical composition of the surface (B), or physical structure of the surface can both lead to an increased WCA, in accordance with the Cassie-Baxter equation. Surface D exhibits the highest WCA, as it combines a low surface energy material with a physical structure that can trap substantial quantities of air in the surface. Examples of plant and insect surfaces belonging to each of these surface types are presented in the inset images. The inset images were reproduced with permission from Koch *et al.*, [27] © The Royal Society, 2009.

According to the Cassie-Baxter equation, any variation in the chemical composition or the topographical structure of a surface will affect the values of *θ*_1_ and *f*_1_ respectively, which will result in a change in the observed composite water contact angle (Figure 1). The surface presented in (B) possesses a different chemical composition to that of (A) and exhibits a higher water contact angle *θ*_1_. The surface presented in (C) is composed of the same material as (A), however the altered surface topography enables it to trap a greater quantity of air, decreasing *f*_1_, and causing the observed angle to approach that of air, *i.e.*, 180°. Surface (D) is composed of a low energy material in addition to possessing the Cassie-Baxter structure, which results in a surface that exhibits an even greater water contact angle.

## 2. Cuticular Layer of Insects and Plants

Insects first evolved the ability to fly at least 400 million years ago [28], whilst plants first moved from the water onto the land between 480–360 million years ago [29]. These are the two important evolutionary events in the history of life because they coincided with changes in the global environment at the time. Despite their differences in nature, both (plants and insects) have developed a cuticle layer that covers all tissues that are exposed to the external surroundings in order to cope with environmental stresses. This layer is secreted by a single layer of epidermal cells, forming a lipophilic structure [30,31,32,33,34,35,36,37]. This structure consisted of two major components which can be distinguished by their solubility in organic solvent [38]. One of them, the cuticular wax component, is monomeric and can be extracted by organic solvents whilst the second component is polymeric and cannot be extracted [38]. Even though the skeletal structures of the two surfaces are very similar, their building materials have some differences. Both plant and insect waxes are consisted of mixture of aliphatic hydrocarbons and their derivatives that contain one or more oxygen functional groups including esters, ketones, alcohols, aldehydes and fatty acids [31,39]. Insect hydrocarbons, however, may also include saturated, unsaturated and methyl-branched hydrocarbons [35,40] whilst plant waxes may contain secondary metabolites such as triterpenoids, phenylpropanoids, and flavonoids [31]. The polymeric compartment is called “cutin” in plants which is made of mainly of ω- and mid-chain hydroxyl and epoxy C_16_ and C_18_ fatty acids as well as glycerol [31], whilst in insects it is a mixture of chitin (poly-*N*-acetylglucosamine) and protein [32,33,41].

The cuticle can serve various purposes, which depend on the nature and living conditions of the organism. Beetles that live in deserts have developed their surface structure to enable them to collect water from fogs for later consumption [42]; water striders with hair-like features enable them to “skate” across the surface of water without penetrating it [43]; colonizing insects, e.g., termites, use their surface chemistry as a tool for communication between individuals [44,45]. Plants in the genus *Nepenthes* have various waxes covering their surfaces which enable them to capture insects as sources of nutrients [46]. In many plant species, the cuticle also acts to reflect visible light and can absorb UV radiation to protect against sun damage [47,48]. The presence of these components can also reduce adhesion of any particles coming into contact with the surface [49,50]. In order to produce such a sophisticated surface, the cuticular waxes are transported onto the surfaces where they self-organize into a smooth two dimensional (2D) wax film or three dimensional (3D) wax crystals, which afford the surface the specific properties required. One important function of these natural surfaces is that they are superhydrophobic and self-cleaning. In subsequent sections, we will discuss some of the major topologies adopted by the cuticular waxes present on plant leaves and insects. In addition, their corresponding chemical composition will also be discussed. Approximately 270,000 plant species [51] and more than 1 million insect species [28] have been described to date; as a result, this review will focus on a number of the important and more relevant surfaces. Much additional information regarding plant waxes can be found in Koch *et al.* [51] which is a dedicated review of plant surfaces. This review will focus on the comparison between the structure, composition and properties of plant leaves and insects, as the two main groups of organisms exhibiting superhydrophobicity and self-cleaning.

### 2.1. Surface Morphology of Insect Wings

**Figure 2:**
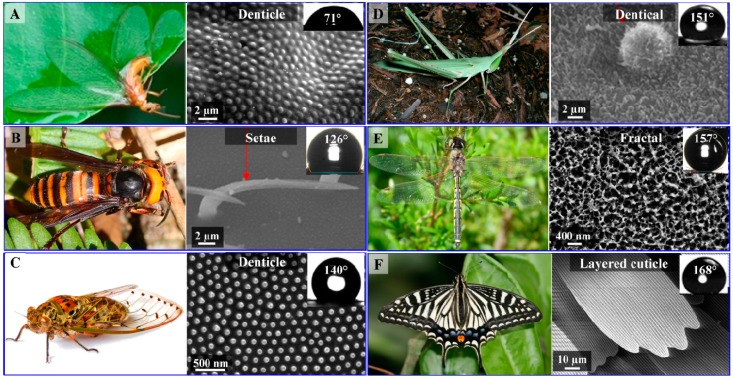
Photographs and SEM images of the surfaces of insect wings. Inset images in electron micrographs depict water droplets on the surfaces of the wings, annotated with their respective WCA. (**A**) *Isoptera Schedorhinotermes* sp.; (**B**) *Hymenoptera Vespa* sp.; (**C**) *Hemiptera Meimuna microdon*; (**D**) *Orthoptera Acrida cinerea cinerea*; (**E**) *Odonata Hemicordulia tau*, and (**F**) *Lepidoptera Papilio xuthus*. Photographs B, D, E © Encyclopedia of Life; image F © Stepanka Nemcova and Anne Ten Donkelaar. Micrographs B and D were adapted with permission from Byun *et al.*, [62]; © Elsevier, 2009. Image A and micrograph C were adapted with permission from Watson *et al.* [63]; © PLoS, 2011.

Insect wing membranes are composed of lightweight building materials with a thickness ranging from 0.5 µm to approximately 1 mm [52]. Their wings are framed by a system of veins which aid in stabilizing the wing as a whole [30,53,54]. The basic framework consists of chitin in the form of a long chain crystalline polymer providing support for the membrane and bearing the forces applied to the wings during flight [55,56]. The junctions between the vein and the wing membrane are comprised of resilin which enhances the flexibility of the wing [57,58,59]. The venation system, together with their light-weight building materials supports routine flights as well as longer colonization flights [45,60,61]. In order to minimize their mass but still retain the ability to protect themselves from wetting and pollutants, insect wing surfaces display a highly-ordered, rough structure, composed of numerous micro- and nanometer-scale features. A systematic terminology to describe the 2D and 3D micro- and nano-scale structures of the insect cuticle has not thus far been developed, so in this review we will use the description of the structures given by authors, imaged using scanning electron microscopy (SEM) and highlight the most relevant morphologies of the insect wings (Figure 2).

Photographs and SEM images of six different insect species belonging to six different Orders are presented in Figure 2. “Setae”, “denticles” and “fractal” are terms that are usually used to describe the features of the wax crystals present on surfaces of the wings. Setae appear as high aspect ratio needles or hairs, denticle structures can vary in morphology from small hemispheres to taller pillars and fractal structures are composed of nanoscale irregular fine protrusions [62]. Wood termite (*Schedorhinotermes* sp.) and cicada (*Meimuna microdon*) wings are covered by a single layer of denticle structures, whilst the wings of the hornet (*Vespa* sp.) appear to be covered by multiple setae (Figure 1). Both structures exhibit water contact angles (WCA) less than 150° [63,64,65] and are therefore not classified as superhydrophobic. Conversely, the surface of the grasshopper (*Acrida cinerea cinerea*), dragonfly (*Hemicordulia tau*) and butterfly species (*Papilio xuthus*) wing exhibited a WCA greater than 150°. The data presented in Table 1 summarizes the morphologies and WCA of some insect species. It can be seen that most species that possess surfaces with setae structures do not exhibit superhydrophobicity, whereas species with more sophisticated fractal and layered cuticle patterns do commonly possess superhydrophobic properties. These types of structures appeared to form more than one level of structure, *i.e.*, be composed of a hierarchical structure, suggesting that hierarchical structures may increase the hydrophobicity of the surface.

**Table 1:** Micro-scale and nano-scale wax crystals on epidermal cells of insect wing surfaces and their WCA ^(a)^.

Order	Species	Structural Morphology	WCA (°)
*Coleoptera*	*Allomyrina dichotoma*	Setae	54
*Coleoptera*	*Chrysolina virgata*	Setae	71
*Isoptera*	*Schedorhinotermes sp.*	Setae	71
*Coleoptera*	*Amphizoa sinica*	Setae	109
*Hymenoptera*	*Vespa simillima xanthoptera*	Setae	121
*Hymenoptera*	*Vespa dybowskii*	Setae	126
*Hemiptera*	*Meimuna microdon*	Denticle	140
*Orthoptera*	*Atractomorpha lata*	Denticle	148
*Orthoptera*	*Acrida cinerea cinerea*	Denticle	151
*Odonata*	*Hemicordulia tau*	Fractal	157
*Odonata*	*Hemianax papuensis*	Fractal	161
*Lepidoptera*	*Artogeia canidia*	Layered cuticle	162
*Lepidoptera*	*Papilio xuthus*	Layered cuticle	168

^(a)^ This table was modified and updated from Byun *et al.* [61]. Terminology regarding structural morphology was also adopted from Byun *et al.*

The effect of surface roughness on wetting properties has been well established, as well as the fact that the mechanism of deactivation of CI is scale-dependent [66,67,68]. The presence of multiscale/hierarchical structures, together with the hydrophobicity of the components making up a surface are key factors in determining the superhydrophobicity of a surface. Nosonovsky and Bhushan calculated the optimal surface requirements to establish superhydrophobicity; these factors are consistent with those described by the Cassie-Baxter model, which applies to many superhydrophobic surfaces possessing hierarchical structures [25,69].

### 2.2. Plant Surface Structures

**Figure 3:**
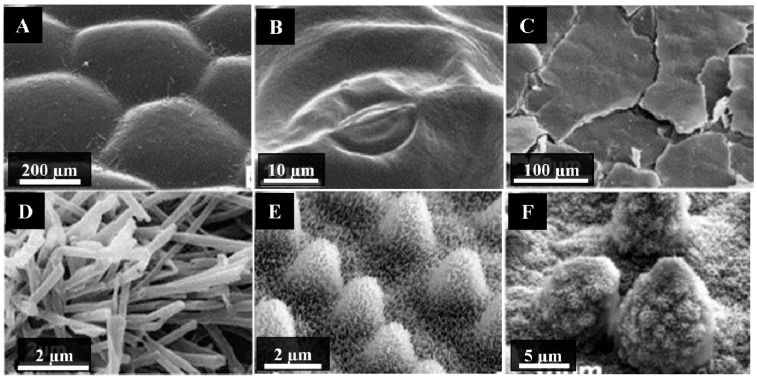
Scanning electron micrographs of the cuticular waxes of plant leaves. (**A**) *Anubias barferi*; (**B**) *Hydrocotyle bonariensis*; (**C**) *Crassula ovate*; (**D**) *Thalictrum flavum glaucum*; (**E**) *Oryza sativa*, and (**F**) *Nelumbo nucifera*. Adapted with permission from Koch *et al.*, [51] © Elsevier, 2009.

Twenty three different wax types have been identified, which contain differences in their orientation and the patterns formed on the surface. Their classification is based on their chemical and morphological features, a description of which can be found in Barthlott *et al.* [70]. A large variation in the morphologies of epicuticular waxes has been identified, with their size ranging from 0.5 to 100 µm. An excellent overview of the terminology used to describe the waxes, together with a description of their micromorphology is provided by Barthlott *et al.* [70] and Jeffree [71]. Some examples are shown in Figure 3.

Neinhuis and Barthlott reported the static WCA of 200 water repellent plant species [26]. Most of these plants were classified as superhydrophobic, as they exhibited a WCA greater than 150°. The common feature shared by these surfaces is that they each possess a very dense arrangement of three-dimensional cuticular wax crystals on their surface, oriented such that they have a hierarchical surface or “Cassie-Baxter structure”. Not all plant surfaces have cuticular structures that enhance the wettability of the surface, however. One such example is *A. barferi*, a plant commonly used in aquariums (Figure 3). Its surface appears relatively smooth under microscopic analysis. Other examples of leaf surfaces that appear smooth are *Hydrocotyle bonariensis* (coastal pennywort) and *Crassula ovate* (jade plant). Their surfaces comprise smooth, 2D wax films without 3D wax crystals evident on the surface. When placed in contact with water, their surfaces tend to conform to the Wenzel wetting state [27]. Conversely, the surfaces of *Thalictrum flavum glaucum* (yellow meadow rue), *Oryza sativa* (rice) and *Nelumbo nucifera* (lotus) contain dense layers of microscale and sub-micron scale features. Each of these surfaces is well-known for their superhydrophobic characteristics. These surfaces conform to the Cassie-Baxter wettability model and are able to maintain a stable superhydrophobic state over time.

## 3. Correlation between the Chemistry and Morphology of Cuticular Waxes

Despite their differences, insect wings and plant leaves possess certain similarities. Their surface morphologies appear very different (Figure 2 and Figure 3); however they serve a similar purpose. Those surfaces that exhibit superhydrophobic and self-cleaning properties, whether they are insects or plant leaves, contain a layer of surface hierarchy together with an appropriate surface chemistry. In the plant literature it has been established that the dominating chemical compounds belong to commonly encountered wax types, a summary of which is presented in Table 2.

**Table 2:** The chemical composition of common plant leaves wax types ^(a)^.

Species	Structural Morphology	Major Compounds	CA (°)
*Crassula ovate*	Crust	Aldehydes C_30_, C_32_; alkane C_31_	NA ^(b)^
*Eucalyptus* sp.	Di-ketone tubules	β-diketone C_33_	162
*Magnolia grandiflora*	Films	Fatty acids C_24_-C_30_, prim. Alcohols C_24_-C_28_	NA
*N. nucifera*	Nonacosanol tubules	Sec. alkane-diol C_29_	162
*Thalictrum* sp.	Nonacosanol tubules		154
*Oryza sativa*	Platelets	Aldehydes	>150
*Aristolochia* sp.	Transversely ridged rodlets	Ketones; alkanes C_31_	160

^(a)^ References for the surface chemistry and wax types are listed in [72]. The morphologies presented in Table 2 are shown in Figure 3; ^(b)^ NA—Not available.

The type of wax, together with its orientation on the surface has been found to be characteristic for certain plant groups. As a result, these characteristic combinations of chemical composition and orientation have been used in plant taxonomy [70,73,74]. The surface wax morphologies can range from being very irregular to highly organized, which begs the question as to what controlling factors are present to determine the three dimensional structure of the surface wax crystals. There exists a lack of information on this matter, however it has been assumed that the cutin network of the leaf may play a role in controlling the orientation of the crystals [75].

Whilst great efforts have been made to understand the functionality and mechanisms responsible for creating the plant cuticular waxes, there is a paucity of information that correlates the wettability and chemistry of the surface of insects. To date, only one study has reported the precise chemical composition of insect cuticles, together with their corresponding wettability [76]. The surface chemistry present on the surface of dragonfly wings (*Odonata Hemicordulia tau*) is responsible for their “pillar-like” structure, which significantly influences their surface wettability, exhibiting a WCA of 157° [76,77]. The specific compound or compounds responsible for forming the dragonfly wing structures was not determined; however approximately 80% of the surface was composed of aliphatic hydrocarbons, including saturated and methyl branched alkanes. The commonly encountered surface chemistry of some insects, together with their corresponding water contact angles are given in Table 3.

**Table 3:** Wax components present in insect wing cuticles.

Order	Species	Major Compounds	WCA (°)	References
*Coleoptera*	*Leptinotarsa decemlineata*	C_25_-C_53_ straight & branched alkane (dimethyl branch)	NA ^(a)^	[78]
*Coleoptera*	*Zygogramma exclamationis*	C_23_-C_56_ alkanes and alkenes		[79]
*Isoptera*	*Zootermopsis nevadensis*	C_19_-C_41_ straight and branched alkanes	NA	[80,81]
*Isoptera*	*Schedorhinotermes sp.*	NA	71	[64]
*Orthoptera*	*Habrobracon hebetor*	Homologous series of n-alkanes, 11-, 13-, and 15-methyl alkanes, 13,17-dimethyl alkanes, and Z-5, Z-7, and Z-9-alkenes	NA	[82]
*Hymenoptera*	*Muscidifurax* sp.	Methyl alkane C_29_-C_39_	NA	[83]
*Odonata*	*Hemicordulia tau*	86% C_10_-C_34_ straight and branched alkanes, 14% hexadecanoic acid	157	[76]
*Odonata*	*Hemianax papuensis*	38% C_14_-C_30_ straight and branched alkanes, 38% carboxylic acid	161	[84]

^(a)^ NA—Not available.

The common chemical composition of the cuticle of insects appears to be aliphatic hydrocarbons, however as there is still a lack of information regarding the wettability of these surfaces and hence little, if any, comment can be made regarding the correlation between these two characteristics. Examining the information contained in Table 2 (plants) and Table 3 (insects), it can be seen that the surfaces of plant leaves contain a greater variety of compounds, particularly those containing oxygen *(i.e.*, alcohols, aldehydes, ketones and fatty acids). It can then be concluded that the surfaces of plant leaves appear to contain a greater proportion of polar compounds than that of insect cuticles. The compositions of the cuticular waxes of plants appear to be similar between those that exhibit superhydrophic and those that exhibit hydrophilic characteristics. In the case of insects, given the lack of information on the correlation between surface chemistry and surface topography, no conclusions can yet be drawn; however it is certain that for materials with similar hydrophobicity, the surface morphology will be the key factor in determining their wettability.

## 4. Summary and Outlook

Nature has provided an excellent selection of valuable templates for the fabrication of artificial surfaces with unique micro/nano-scale properties for engineering and other technological applications [71,85]. Various techniques have been developed and applied in order to produce synthetic surfaces that possess desired characteristics, including superhydrophobicity and self-cleaning properties, as these have great potential impact, particularly in the area of biomedical implants. A great deal of research has recently been performed in an effort to understand the mechanism of action of these surfaces in order to optimize their applicability [15,85,86]. As emphasized throughout this review, natural plant and insect surfaces that possess Cassie-Baxter characteristics represent the most promising templates for synthetic biomimetic substrata.

**Figure 4:**
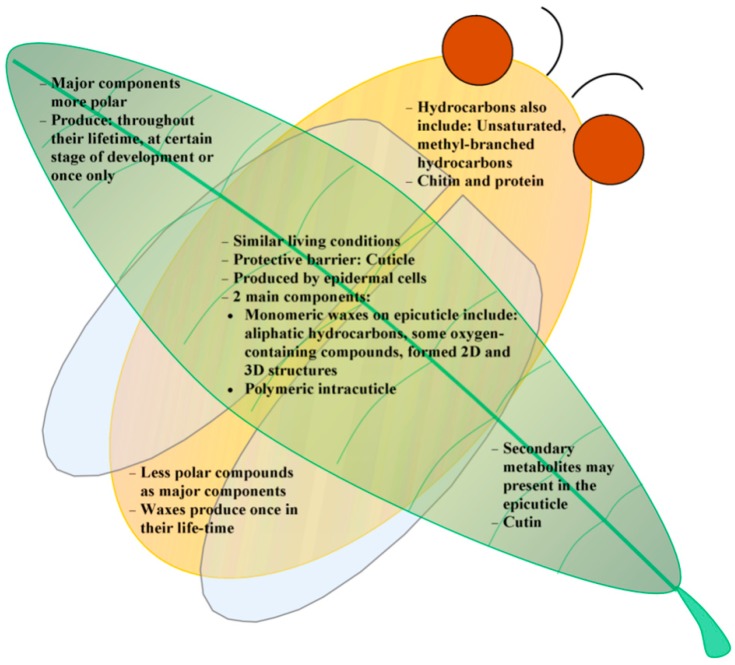
Summary of similarities and differences between the surface characteristics of insect and plant cuticles.

The diversity in wax crystal sizes and shapes of the surface structures present on insects and plants reflects the systematic relationships that exist among species [87,88]. Investigation of the micro/nano-scale structures present on these surfaces may allow species within the same order to be distinguished or taxonomically classified. The scientific community has a long history of studying the mechanisms by which individual organisms communicate; it has been shown that cuticular chemistries play an important role in social communication amongst insects. These roles include determination of colony membership, hierarchical dominance, fertility status and task group membership [45,89,90,91,92,93]. In plants, cuticular chemistry plays a role in determining host/pest relationships [73,92,94,95]. The similarities and differences that exist between insect and plant surfaces are summarized in Figure 4.

Insects are directly associated with food production and health, and as a result their chemical characteristics have been studied over a long time as part of the agriculture sciences. Wettability on the other hand has not really been addressed until very recently, and there is still a lack of information in this specific field. Few researchers have simultaneously investigated the surface chemistry and topography of insect wings in the context of their wettability. As a result, a large gap has emerged in the available knowledge pertaining to the correlation between the surface structure and surface chemistry of insect surfaces, which has been well established for plant cuticles. Therefore, more research needs to be conducted that will provide a greater insight into the correlation between surface chemistry, surface topography and surface wettability. Moreover, in light of their superhydrophobic, self-cleaning, and recently discovered bactericidal activities, new research directions are opening for the development of bacteria-resistant technologies [77,96,97,98]. 
